# Using Soil Apparent Electrical Conductivity to Optimize Sampling of Soil Penetration Resistance and to Improve the Estimations of Spatial Patterns of Soil Compaction

**DOI:** 10.1155/2014/269480

**Published:** 2014-12-31

**Authors:** Glécio Machado Siqueira, Jorge Dafonte Dafonte, Javier Bueno Lema, Montserrat Valcárcel Armesto, Ênio Farias França e Silva

**Affiliations:** ^1^Centro de Ciências Agrárias e Ambientais, Universidade Federal do Maranhão, BR-222, KM 04, s/n, Boa Vista, 65500-000 Chapadinha, MA, Brazil; ^2^Escuela Politécnica Superior, Universidad de Santiago de Compostela (USC), Campus Universitario, 27002 Lugo, Spain; ^3^Departamento de Engenharia Agrícola, Universidade Federal Rural de Pernambuco (UFPRE), Rua D. Manuel de Medeiros, s/n, 52171-900 Recife, PE, Brazil

## Abstract

This study presents a combined application of an EM38DD for assessing soil apparent electrical conductivity (EC_a_) and a dual-sensor vertical penetrometer Veris-3000 for measuring soil electrical conductivity (EC_veris_) and soil resistance to penetration (PR). The measurements were made at a 6 ha field cropped with forage maize under no-tillage after sowing and located in Northwestern Spain. The objective was to use data from EC_a_ for improving the estimation of soil PR. First, data of EC_a_ were used to determine the optimized sampling scheme of the soil PR in 40 points. Then, correlation analysis showed a significant negative relationship between soil PR and EC_a_, ranging from −0.36 to −0.70 for the studied soil layers. The spatial dependence of soil PR was best described by spherical models in most soil layers. However, below 0.50 m the spatial pattern of soil PR showed pure nugget effect, which could be due to the limited number of PR data used in these layers as the values of this parameter often were above the range measured by our equipment (5.5 MPa). The use of EC_a_ as secondary variable slightly improved the estimation of PR by universal cokriging, when compared with kriging.

## 1. Introduction

Soil physical properties play an important role on crop growth, if not the most important [1]. On the other hand, soil cultivation under different land uses may cause changes in soil spatial variability, depending on tillage intensity [2].

The use of farm machinery in agricultural production systems disturbs the soil structure and often may generate soil compacted layers that affect soil aeration and infiltration capacity. Different soil management systems produce different levels of soil compaction, depending on water content, type of soil, and agricultural machinery operations.

Soil penetration resistance has been found to be well correlated with root growth, and these two variables are inversely proportional. When soil water content decreases, soil mechanical resistance increases, because of the diminution of cohesion within the solid fraction of soil [3–5]. Several authors have shown that root growth can be restricted or even impeded when PR values vary between 1.0 and 3.5 MPa [6, 7]; however others quoted threshold between 2.0 and 4.0 MPa [8] for limitations to root grow.

Hill and Meza-Montalvo [9] concluded that agricultural machinery traffic during the crop growth cycle may increase the values of soil density and soil resistance to penetration to 50%. For this reason, the quantification of soil PR changes caused by soil management is an important parameter for maintaining desirable levels of production and environmental sustainability.

Most farmers consider the soil as uniform for its management, but soil properties are variable in space and time. As a result of these variations, the use of the average value of a soil property could lead to wrong management decisions. This notwithstanding, conventional agriculture has been based on soil sampling with few samples [10].

The electrical conductivity (EC) is the property that has a material to transmit or conduct electrical current [11–14]. The apparent soil electrical conductivity (EC_a_) is a measure of the bulk electrical conductivity of the soil and is influenced by various factors such as soil porosity, concentration of dissolved electrolytes, texture, quantity and composition of colloids, organic matter, and water content in the soil [11]. Recent research found that apparent soil electrical conductivity measurements using electromagnetic sensors can be used to make rapid measurements of soil water content, soil clay content, cation exchange capacity and levels of exchangeable calcium and magnesium, depth of horizons with a “pan” caused by compaction, organic matter content, and salt content in soil solution [14]. In this way, measurements of apparent soil electrical conductivity (EC_a_) can be used to define specific management zones.

Rhoades et al. [11] and Nadler [12] claim that soils with high water content have a higher value of EC_a_, which makes the interpretation of EC_a_ data difficult. This is because the water content varies with depth, even if the soil is uniform, which may cause strong EC_a_ variations as a function of depth. Moreover, temperature also affects the soil EC_a_ [11, 15]. This is because increased soil temperature has effect on water viscosity and therefore affects the mobility of dissolved electrolytes into the soil solution [16]. Summarizing, it is widely accepted that the main factors that influence EC_a_ are texture, soil water content, and salinity [14, 17–19]; there is good correlation between these soil properties and EC_a_ [20].

The aim of this study was to use apparent soil electrical conductivity data (EC_a_), measured by EM38, first to generate optimal soil sampling designs and then to improve the spatial estimation of soil resistance to penetration (PR) on the studied field.

## 2. Material and Methods

The studied field is 6 ha in surface, and it is located at Castro Riberas de Lea, Lugo, Northwestern Spain. The geographical coordinates are 43°09′49′′N and 7°29′47′′W, average elevation is 410 m and average slope is 2%. The soil of the study area is classified according to FAO-ISRIC [21] as Gleyc Cambisol. At the sampling date, the field was cropped with maize for silage under no tillage; before maize, the field was devoted to permanent grassland for silage. A more thorough description of this area can be found in Siqueira et al. [22].

**Figure 1:**
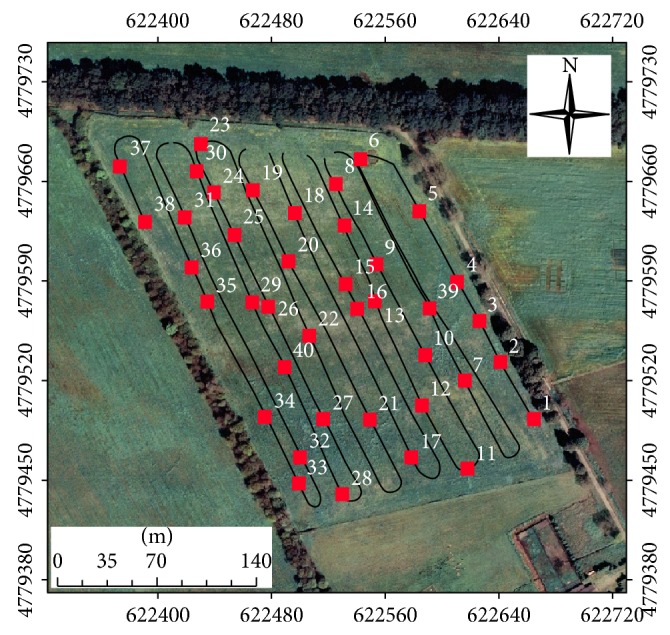
Continuous record of soil apparent electrical conductivity (EC_a_) obtained by electromagnetic induction (line) and the optimized sampling scheme consisting 40 points (circles).

The apparent soil electrical conductivity (EC_a_) was measured with an induction electromagnetic device, namely, EM38-DD (Geonics Limited). The EM38-DD is constructed by mechanically and electrically integrating two standard EM38 ground conductivity meters. The bottom instrument's transmitter-receiver dipoles are oriented parallel to the earth in horizontal dipole (EC_a_-H), while for the top instrument, which controls the digital output of the whole instrument, the dipoles are oriented perpendicular to the earth surface in vertical dipole (EC_a_-V). In the EC_a_-V mode, the primary magnetic field can effectively penetrate to a depth of 1.5 m, while the EC_a_-H mode is effective for shallower investigation (0.75 m) [12]. The data were collected on 23/6/2008 in 1859 points (Figure 1), using EM38DD a field computer and a GPS RTK to georeference EC_a_ data.

The EC_a_ data, together with the software ESAP-RSSD 2.35 [23], were used to identify the 40 optimal locations to perform measurements with the VERIS P3000 equipment (Figure 1). The penetrometer used in this research was the Profiler 3000, manufactured by Veris Technologies Inc. It was a self-contained, trailer mounted device, designed to be pulled through the field by a vehicle [24]. An onboard power unit and hydraulic cylinder were used to insert the penetrometer to a maximum depth of approximately 90 cm. Maximum insertion force was limited to approximately 5.5 MPa with the sensing tip, to prevent overload of the mechanical components and sensing system. A second hydraulic cylinder pivoted the penetrometer mast through a transverse arc, allowing approximately 90 cm of side-to-side displacement for acquiring data across in row and between row locations. Data collection was triggered every 2 cm. Soil electrical conductivity (EC_veris_) was sensed immediately above the penetrometer tip. The penetrometer tip itself was electrically insulated from the penetrometer shaft with a thin dielectric ring. Electrical contact with the tip was by means of a small steel rod inside and insulated from the hollow shaft.

**Figure 2:**
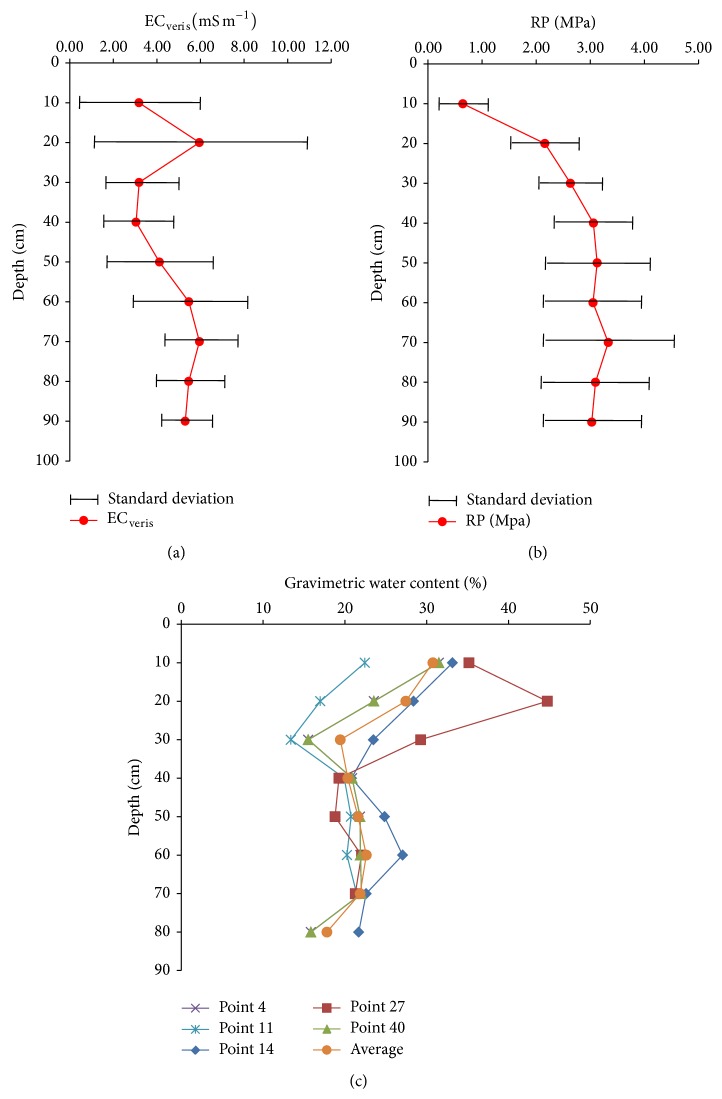
Mean values of EC_veris_ measured continuously, penetration resistance (PR) measured at 40 points, and gravimetric water content at five selected points.

In this study the soil penetration resistance data was grouped into the following layers: 0.0-0.1 m (PR_0.0-0.1_), 0.1-0.2 m (PR_0.1-0.2_), 0.2-0.3 m (PR_0.2-0.3_), 0.3-0.4 m (PR_0.3-0.4_), 0.4-0.5 m (PR_0.4-0.5_), 0.5-0.6 m (PR_0.5-0.6_), 0.6-0.7 m (PR_0.6-0.7_), 0.7-0.8 m (PR_0.7-0.8_), and 0.8-0.9 m (PR_0.8-0.9_) (Figure 2). In each measurement location, the PR and EC_veris_ profile was measured in six near points. Therefore, the PR and EC_veris_ data showed for each location is the mean of the six profiles measured (Figures 2(a) and 2(b)).

Gravimetric soil water content was measured at these sampling points: 4, 11, 14, 27, and 40 (Figure 2(c)), in order to relate the EC_a_ values with the soil water content. Pearson's coefficients of correlation and significance levels were calculated between the data using pairs with the package “hmisc” [25].

The geostatistical analysis included preliminary statistical analysis, Kolmogorov-Smirnov normality test, analysis of trend, variogram modeling, and estimating values for unsampled locations using the kriging interpolation technique. Initial analysis showed that some variables had a trend; in this case the residual ordinary kriging was used.

Using geostatistics, there are two options to solve the problem created by the existence of a drift within the neighborhood search. On the one hand, it can be assumed that the drift is an equation with constant coefficients for the entire study area, which leads to residual kriging. On the other hand it can be assumed that coefficients of the drift equation vary by location of the study area, which results in universal kriging or kriging with trend model; this procedure simultaneously solves drift while kriging equations are solved. In this research the residual ordinary kriging was used (Table 3). The residual variograms were fitted to variogram models with cross validation using the package Gstat for R [26]; Surfer 7.0 software was used for the creation of the maps.

Some of the variables with a trend were interpolated by universal cokriging [27], instead of ordinary cokriging or cokriging with external drift. The software used to perform ordinary kriging, universal kriging, and universal cokriging was Gstat for R [26]. Using cokriging the covariance matrix must be positive definite [27–29].

## 3. Results and Discussion

Table 1 shows the statistical parameters for the apparent electrical conductivity (EC_a_) measured with EM38-DD and soil resistance to penetration (PR) and electrical conductivity (EC_veris_) measured with penetrometer Veris P3000 (Table 4). All studied properties showed log-normal distribution with statistical Kolmogorov-Smirnov test with significance level of 0.01.

**Figure 3:**
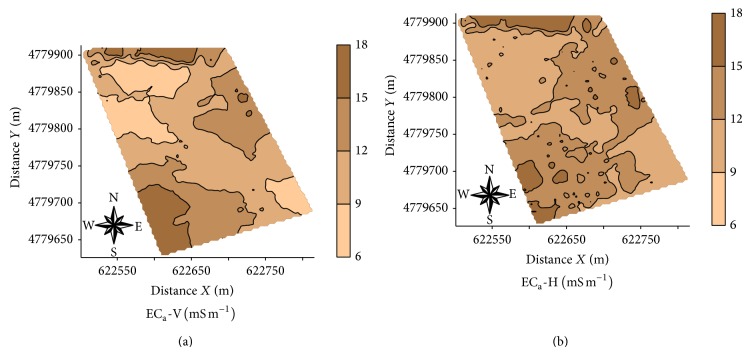
Maps of soil apparent electrical conductivity (EC_a_-V and EC_a_-H).

Coefficient of variation of the studied properties had a moderate variability with values between 12 and 60%, according to the classification of Warrick and Nielsen [1]. The following data sets had high CV values (>60%): EC_veris  0-0.1_; EC_veris  0.1-0.2_; EC_veris  average  0.0–0.4_ y PR_0-0.1_. The presence of high values of CV mainly for electrical conductivity (EC_veris_) were expected since the number of measurements was much lower (maximum 140), compared to EC_a_-V and EC_a_-H data measured with the EM38-DD device (1859 measurements, Figure 3).

The small number of measurements obtained with Veris P3000 at the deepest soil layers (Table 1) was mainly because the equipment has a safety valve that prevents the measurement of soil PR above 5.5 MPa. The presence of a compacted layer or gravel in the soil profile was the main reason for because reducing the number of measurements at the deepest layers. Figure 2 shows the average values of the electrical conductivity (EC_veris_, Figure 2(a)), penetration resistance (PR, Figure 2(b)), and gravimetric water content (%, Figure 2(c)) in the field studied.

The average values of the electrical conductivity measured with Veris penetrometer (EC_veris_) showed an increase with increasing depth, which is in accordance with results presented by Johnson et al. [30] and Motavalli et al. [31]. Increasing values of water content and soil clay content in depth, contributed to increased values of EC_veris_ [32, 33]. The mean values of soil resistance to penetration (PR) increased in depth, but there was a slight decrease in the values of PR in depth below 0.5 m depth, because of a gravel layer located at this depth.

Figure 2(a) shows that the standard deviation of EC_veris_ data is higher in the surface layers, decreasing in the deeper layers, the opposite occurs with PR data because in depth the stone volume is larger causing PR values exceeding in many cases the equipment measurement limit of 5.5 MPa, as reflected in the smaller number of measurements in these layers of soil (Table 1).

Gravimetric soil moisture was measured at locations 4, 11, 14, 27, and 40 (Figure 2(c)); the choice of these locations was made on the basis of the topography of the area; these locations are representative for spatial variability of water content. It is seen that in the surface layers the water content varies more than in the deeper layers. The topsoil has higher water content than in the deeper layers in the sampling date.

In Figure 2(a) it can be seen that the graph of EC_a_ is very similar to the soil water content graph (%, Figure 2(c)). Thus, EC_veris_ dataset obtained by the penetrometer Veris is an indirect way to obtain information about the soil water status, facilitating the interpretation of PR data, because the direct measurement of the volumetric water content of the soil is hard and slow, particularly in this type of soil with a high (>370,00 g kg^−1^) amount of stones [34]. Motavalli et al. [31] studied the use of equipment Veris P3000 to detect the effects of compaction, and they found that the values of EC_a_ were correlated with soil compaction and clay content in the soil profile, thereby enabling to relate EC_a_ with PR.

**Figure 4:**
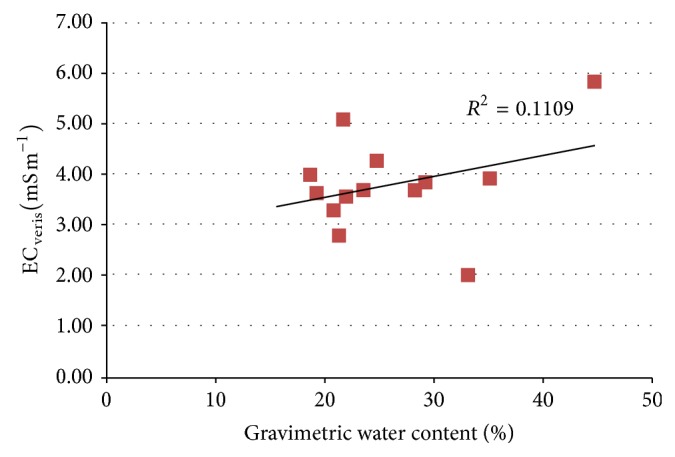
Correlation between EC_veris_ and gravimetric soil water content.

Figure 4 shows the relationship between soil water content and EC_veris_ for the locations where soil water content was measured. It is apparent that there is no good correlation between EC_veris_ and gravimetric soil water for the selected points; to assess dependence between these two variables, more soil water content measurements would be needed.

Sudduth et al. [33] comparing the penetrometer Veris P3000 with ASAE Standard penetrometer found no significant differences between the values of soil resistance to penetration (PR) for different penetrometers studied. Canarache [6] showed that in addition to soil moisture, also PR is related to other soil properties such as bulk density fine sand, sand, and clay contents. However, several authors claim that PR values are mainly related to moisture and soil bulk density [35–37]. In our case study, as soil sampling became difficult due to increasing gravel content in depth, PR measurements were essential to assess the physical status of the soil.

Summarizing, we showed that the joint use of EC_veris_ and PR data can detect changes in soil density and water content due to compaction, in addition to natural variations in soil texture.

Several authors provided different soil PR threshold values, regarding limitations for crop production. For example, Taylor and Gardner [37] reported that PR values greater than 2 MPa inhibit vegetative growth. Taylor and Burnett [38] studied the development of different crops (*Gossypium hirsutum, Sesamum indicum, Cyamopsis tetragonolobus, Sesbania exaltata, Phaseolus aureus, Vigna sinensis* var. Chinese Red, and* Sorghum vulgare* var. Sumac sorghum) with different tillage systems, these authors describe that values from 2.8 MPa began to restrict the root growth. Ehlers et al. [39] studied root growth of oats (*Avena sativa* L.) in the 0–0.25 m layer and found that root growth ceased when PR reached values between 3.6 and 4.9 MPa. Letey [40] and Bueno et al. [41] noted that soil PR values to higher than 2.0 MPa are restrictive to root growth.

Bennie [42] stated that more important than soil PR is the rate at which changes occur in soil bulk density until critical density values for vegetative growth are achieved. Bueno et al. [41] studied the PR in the 0–0.25 m layer at a field neighboring to our experimental field under no-till and conventional tillage and found PR values between 0.0 and 3.0 MPa depending on soil water content. In general, the mean value of PR for 0–0.4 m layer (PR_average  0.0–0.4_) in this study was about 2.12 MPa. This value was close to the 2 MPa threshold, commonly cited as restrictive for crop growth. PR_average  0.4–0.9_ was 3.40 MPa, exceeding the value of 2 MPa. However, the average soil water content of this layer is more stable over the study field, as shown in Figure 3, whereas the soil moisture content at 0.0–0.4 m layer varied considerably over this field.

Bueno [43] and Amiama [44], studying the PR at 0.0–0.4 m depth in a field near to the area studied here in several years, found similar values of PR, the higher values of PR were found in the depth layers. Amiama [44] found a moderate correlation between PR and soil water content values.

Table 2 shows the correlation matrix between the measured variables. There is a significant correlation between EC_a_ data EM38-DD and EC_veris_, but the correlation s was not significant below 0.6 m depth. Probably, the lack of correlation at the deepest soil layers is related to the small number of sampling points in these layers obtained with the penetrometer Veris; in turn this is the result of the high content of gravel with depth.

Pearson's correlations between EC_a_ and PR show highly significant negative relationships. This is because EC_a_ is very dependent on the soil water content [11, 12, 14]; Hoefer et al. [45] found good correlations between PR and EC_a_ measured with EM38, and they concluded that EM38 survey can be used to detect subplots with an extreme compaction or noncompaction state. According to Ehlers et al. [39] PR is much more influenced by soil water content than by soil bulk density. The electrical conductivity and PR data obtained with Veris P3000 showed negative significant correlation, which matches the negative correlations between EC_a_ and PR. According to Drummond et al. [24] although there are various equipment available for measuring soil resistance to penetration and soil electrical conductivity, the joint measurement of PR and EC_veris_ would allow to characterize the soil not only along the landscape but also in depth. This could contribute not only to the understanding of the spatial distribution of PR and EC_a_, but also to assessing density, texture, and water content in the soil.

**Figure 5:**
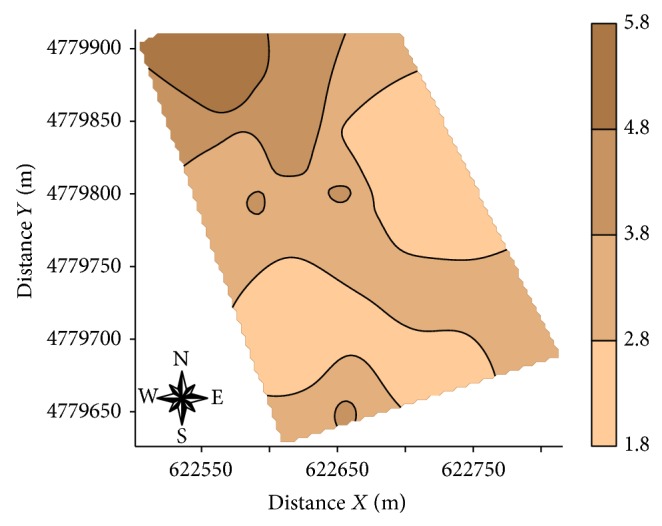
Map of EC_veris  average  0.4–0.9_ (mS·m^−1^) obtained by universal cokriging.

**Figure 6:**
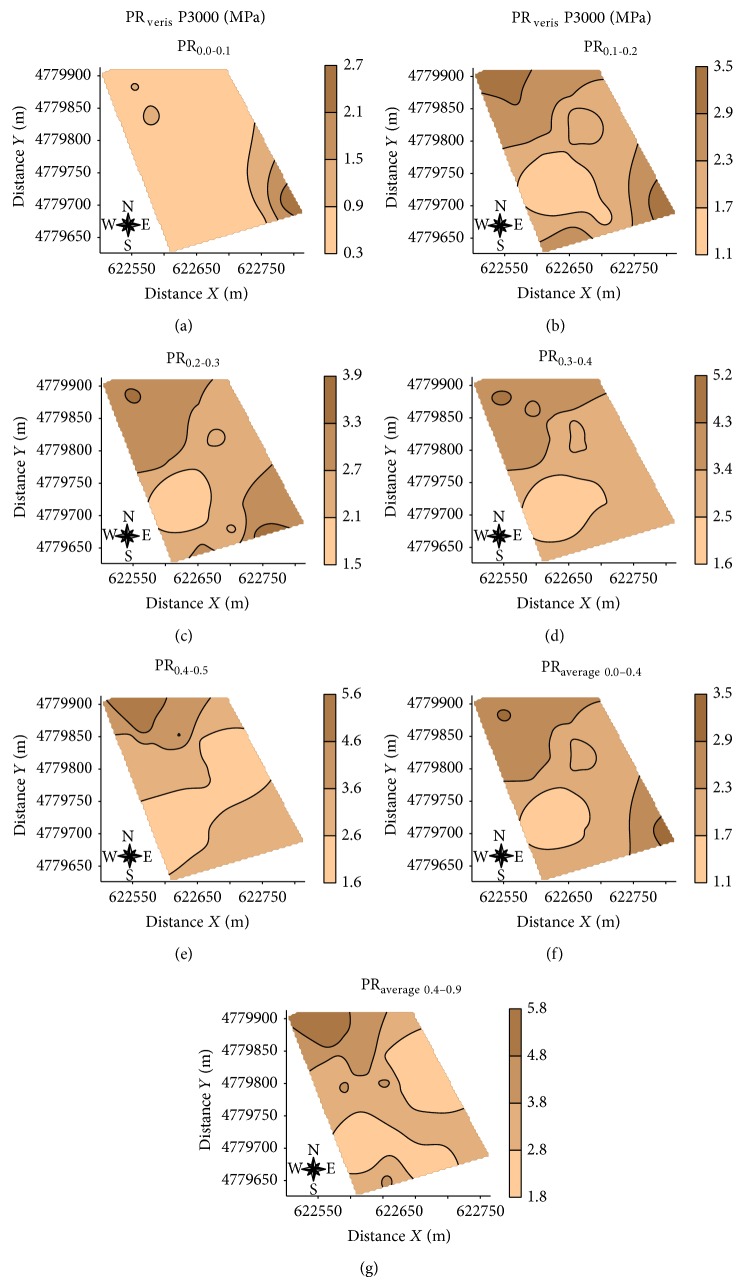
Maps of soil resistance to penetration measured with the penetrometer Veris (PR_0.0-0.1_, PR_0.1-0.2_, PR_0.2-0.3_, PR_0.3-0.4_, PR_0.4-0.5_, PR_0.0–0.4  average_, and PR_average  0.4–0.9_) obtained by universal kriging.

**Figure 7:**
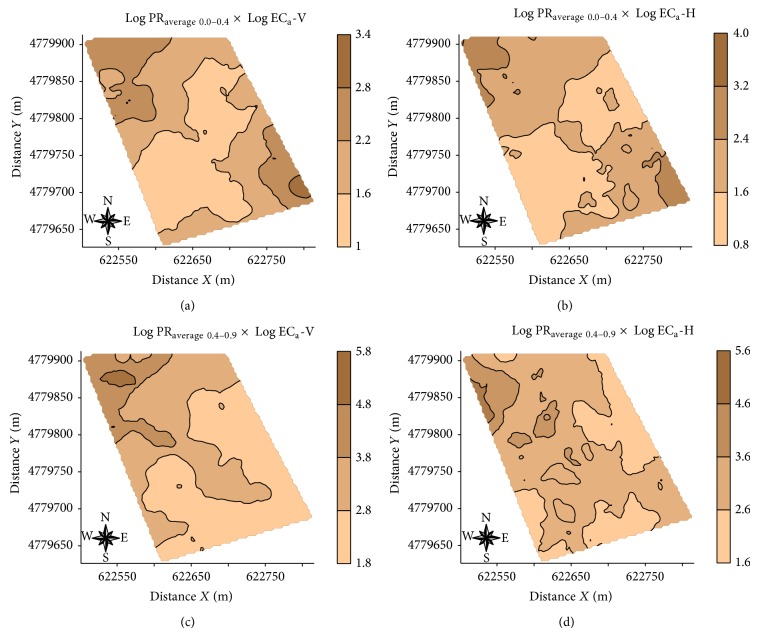
Maps of soil resistance to penetration at the layers 0.0–0.4 m (PR_average  0.0–0.4_) and 0.4–0.9 m (PR_average  0.4–0.9_) obtained by universal cokriging using EC_a_-V and EC_a_-H as secondary variables.

Table 5 shows the type of variogram models fitted to the experimental data and the parameters of these models. EC_veris  0.0-0.1_, EC_veris  0.1-0.2_, EC_veris  0.2-0.3_, EC_veris  0.3-0.4_, EC_veris  0.4-0.5_, EC_veris  0.5-0.6_, EC_veris  0.5-0.6_, EC_veris  0.7-0.8_, EC_veris  0.8-0.9_, EC_veris  average  0.0–0.4_  PR_0.5-0.6_, PR_0.6-0.7_, PR_0.7-0.8_, and PR_0.8-0.9_ showed pure nugget effect. This might indicate that the spacing used between samples was not adequate to detect the spatial variability but may also reflect the small number of points used in the analysis process. The values of the degree of spatial dependence showed that all variables studied had a high value of this parameter, following the accredited criteria of Cambardella et al. [46]. Spherical models were fitted to the experimental variograms of most studied variables (EC_a_-V, EC_a_-H, EC_veris  average  0.4–0.9_, PR_0.0-0.1_, PR_0.1-0.2_, PR_0.2-0.3_, PR_0.3-0.4_, PR_0.4-0.5_, PR_average  0.0–0.4_, and PR_average  0.4–0.9_). The lower range value (a) corresponds to Log EC_veris  average  0.4–0.9_ (50 m), whereas the highest value was found for Log PR_average  0-0.4_ (130 m). Jabro et al. [47] studied the spatial variability of the EC_a_ (mS m^−1^) and PR (MPa) with the Veris penetrometer described values of range for both parameters of 161 m; the values of range in this study were for Veris penetrometer approximately 50 m for EC_veris  average  0.4–0.9_ and 130 m and 70 m for the PR_0.0–0.4_ and PR_average  0.4–0.9_, respectively. Figures 5, 6, and 7, respectively, show the maps for EC_a_-V, EC_a_-H, EC_veris  average  0.4–0.9_, and PR (PR_0.0-0.1_, PR_0.1-0.2_, PR_0.2-0.3_, PR_0.3-0.4_, PR_0.4-0.5_, PR_average  0.0–0.4_, and PR_average  0.4–0.9_).

The map of the EC_veris  average  0.4–0.9_ (Figure 6) has a similar spatial pattern to EC_a_-V and EC_a_-H (Figure 5), exhibiting varying EC in specific areas, in the eastern part of the area that exhibits low EC values in both maps. EC_veris  average  0.4–0.9_ map exhibits areas with higher EC_a_ data, especially in the right center area, this difference in depth of the EC measured with the penetrometer Veris is probably related to the greater water content in depth or with areas where the clay content is increased [11, 12, 14, 19, 47]. We emphasize that the EM38-DD gives a value of EC_a_ it is a measure of the interactions of electromagnetic pulse with a specific volume of soil (water content, density, porosity, clay content, organic matter, etc.) [14] and the penetrometer Veris measures EC in a comparatively small soil volume, providing a better description of the characteristics of the soil in depth, according to what is described by Drummond et al. [24], Sudduth et al. [33], and Jabro et al. [47].

The matrix of linear correlation between the measured data with the EM38-DD equipment (EC_a_-V and EC_a_-H) with EC measured with the penetrometer Veris (EC_a  0.0-0.1_, EC_a  0.1-0.2_, EC_a  0.2-0.3_, EC_a  0.3-0.4_, EC_a  0.4-0.5_, EC_a  0.5-0.6_, EC_a  0.6-0.7_, EC_a  0.7-0.8_, EC_a  0.8-0.9_, EC_a  average  0.0–0.4_, and EC_a  average  0.4–0.9_) (Table 2) confirms the relationship between measures of the EC_a_.

The maps of spatial variability of soil penetration resistance (Figure 7) show that the highest values of PR are located mainly in the northwestern part of the area (PR_0.1-0.2_, PR_0.2-0.3_, PR_0.3-0.4_, PR_0.4-0.5_, PR_average  0.0–0.4_, and PR_average  0.4–0.9_). Maps of PR show the opposite behavior to EC_veris  average  0.4–0.9_ confirming the negative correlation (Figures 5 and 6), where areas with higher EC_a_ showed lower values of PR, corroborating the hypothesis that EC data measured with Veris penetrometer can be used as indicator of soil water content. According to Canarache [6] PR values can be classified according to the degree of constraint on the growth of roots <1.1 MPa very low without limitation for crops; low 1.1–2.5 MPa with little limitation for crops; medium 2.6–5.0 MPa with some limitations for crops; 5.1–10.0 MPa high with severe limitations for crops; 10.1 to 15.0 MPa very high where the roots do not grow virtually; >15.0 MPa extremely high the roots do not grow. In general, the study area is not a problem for root growth, since rarely exceeds the value of 5 MPa PR (PR_0.4-0.5_, PR_0.5-0.6_, and PR_0.6-0.7_) (Table 1) indicating that the study area has medium PR with few limitations for cultivation and some areas with low PR, according to the classification of Canarache [6].

Table 5 shows the fitted parameters of cross variogram models between Log PR_average  0.0–0.4_ × Log EC_a_-V, Log PR_average  0.0–0.4_ × Log EC_a_-H, Log PR_average  0.4–0.9_ × Log EC_a_-V, and Log PR_average  0.4–0.9_ × Log EC_a_-H, since these were the only attributes that showed an improvement in the coefficient of correlation between the measured and estimated values obtained using kriging and cokriging (Table 5).

PR maps made by universal cokriging (Figure 7) are less smooth when compared with PR maps made by ordinary kriging (Figure 6), although the improvement obtained with the use of the estimate cokriging PR has been small.

## 4. Conclusions

The data sets EC_veris_ and PR showed low values of coefficient of variation (CV, %), except for PR at 0.0-0.1 m layer that has a high value of CV (60.30%). The spherical model was fitted to the data of soil resistance penetration (PR_0.0-0.1_, PR_0.1-0.2_, PR_0.2-0.3_, PR_0.3-0.4_, and PR_0.4-0.5_) at the top soil layers. The presence of pure nugget effect for soil penetration resistance at the bottom (PR_0.5-0.6_, PR_0.6-0.7_, PR_0.7-0.8_, and PR_0.8-0.9_) may be caused by the limited number of data available for these layers, since the recorded PR values were often not useful, as they were higher than the maximum value measured by the penetrometer (5.5 MPa). Apparent soil electrical conductivity data (EC_a_) were used to determine the optimized sampling scheme where PR was measured, and this methodology has proved to be efficient and capable for representing the spatial pattern of PR. EC_a_ and PR data show significant negative correlation coefficients, once both properties are related to the soil water content. The use of EC_a_ as secondary variable improved slightly the estimation of PR using universal cokriging with respect to kriging.

## Figures and Tables

**Table 1:** Statistical parameters of the apparent electrical conductivity (EC_a_) measured with EM38, in vertical and horizontal modes, and electrical conductivity (EC_veris_) and soil resistance to penetration (PR) measured with VERIS P3000 at successive layers.

Variable	Units	*N*	Min.	Max.	Mean	Variance	CV	Skewness	Kurtosis	*D*
EC_a_-V	mS m^−1^	1859	4.13	20.13	11.21	6.12	22.07	0.485	−0.243	0.071Ln
EC_a_-H		1859	6.63	20.00	12.12	3.22	14.81	0.839	1.285	0.092Ln
EC_veris 0.0-0.1_		140	1.07	12.27	3.17	7.64	87.00	2.523	6.259	0.317Ln
EC_veris 0.1-0.2_		134	1.58	17.84	5.94	23.48	81.47	1.360	0.798	0.317Ln
EC_veris 0.2-0.3_		131	1.36	8.14	3.18	2.77	52.34	1.562	2.643	0.317Ln
EC_veris 0.3-0.4_		102	1.13	7.79	3.04	2.48	51.88	1.279	1.966	0.317Ln
EC_veris 0.4-0.5_		84	0.62	9.30	4.11	6.02	59.65	−0.179	−1.739	0.330Ln
EC_veris 0.5-0.6_		72	0.80	9.41	5.46	6.76	47.57	−3.680	−10.143	0.361Ln
EC_veris 0.6-0.7_		63	2.79	8.05	5.95	2.83	28.29	−36.860	−197.233	0.404Ln
EC_veris 0.7-0.8_		32	2.13	7.31	5.45	2.49	28.92	−49.662	−418.618	0.432Ln
EC_veris 0.8-0.9_		27	3.17	6.81	5.29	1.27	21.34	−265.192	−7150.197	0.513Ln
EC_veris 0.0–0.4_		507	1.40	9.32	3.83	5.74	62.47	−1.511	−14.054	0.317Ln
EC_veris 0.4–0.9_		278	0.62	7.94	4.51	5.09	49.96	−39.185	−39.185	0.330Ln

PR_0.0-0.1_	MPa	140	0.30	2.47	0.64	0.18	67.30	1.169	1.858	0.317Ln
PR_0.1-0.2_		134	1.10	3.28	2.16	0.40	29.41	−26.517	−121.478	0.317Ln
PR_0.2-0.3_		131	1.49	3.53	2.63	0.32	21.78	−0.423	−0.677	0.317Ln
PR_0.3-0.4_		102	1.68	4.85	3.05	0.53	23.89	0.265	0.025	0.317Ln
PR_0.4-0.5_		84	1.74	5.54	3.12	0.94	31.17	−0.211	4.172	0.330Ln
PR_0.5-0.6_		72	1.92	5.32	3.04	0.86	30.43	−19.154	−60.403	0.381Ln
PR_0.6-0.7_		63	1.60	5.59	3.33	1.46	36.31	−16.549	−88.761	0.404Ln
PR_0.7-0.8_		32	1.54	4.85	3.09	0.99	36.26	−34.572	−285.556	0.432Ln
PR_0.8-0.9_		27	1.62	4.43	3.02	0.87	30.87	−47.972	−495.883	0.449Ln
PR_average 0.0–0.4_		507	1.18	3.06	2.12	0.26	24.05	−48.771	−233.173	0.317Ln
PR_average 0.4–0.9_		278	1.95	5.54	3.40	0.98	29.21	−33.401	−207.070	0.330Ln

*N*: number of measures (maximum six measurements in each location for EC_veris_ and PR); Min.: minimum value; Max.: maximum value; CV: coefficient of variation (%); *D*: normality of the data for test of Kolmogorov-Smirnov (*P* < 0.01, n: normality and Ln: lognormality).

**Table 2:** Pearson's coefficients of correlation matrix between the variables measured by EM38 and VERIS P3000 (^**^significant for *P* < 0.05; ^*^significant for *P* < 0.1).

	EC_a_-V	EC_a_-H	PR_0.0-0.1_	PR_0.1-0.2_	PR_0.2-0.3_	PR_0.3-0.4_	PR_0.4-0.5_	PR_0.5-0.6_	PR_0.6-0.7_	PR_0.7-0.8_	PR_0.8-0.9_	PR_average 0.0–0.4_	PR_average 0.4–0.9_
EC_a_-V	1												
EC_a_-H	0.75^**^	1											
PR_0.0-0.1_	−0.36^*^	−0.44^**^	1										
PR_0.1-0.2_	−0.5^**^	−0.56^**^	0.66^**^	1									
PR_0.2-0.3_	−0.6^**^	−0.59^**^	0.41^**^	0.85^**^	1								
PR_0.3-0.4_	−0.7^**^	−0.53^**^		0.74^**^	0.79^**^	1							
PR_0.4-0.5_	−0.45^**^			0.74^**^	0.74^**^	0.73^**^	1						
PR_0.5-0.6_	−0.52^**^	−0.44^*^		0.51^**^		0.47^*^	0.63^**^	1					
PR_0.6-0.7_				0.61^**^				0.78^**^	1				
PR_0.7-0.8_	−0.49^*^			0.48^*^					0.84^**^	1			
PR_0.8-0.9_	−0.67^**^	−0.62^**^	0.57^*^	0.68^**^					0.79^**^	0.89^**^	1		
PR_average 0.0–0.4_	−0.65^**^	−0.62^**^	0.64^**^	0.95^**^	0.91^**^	0.87^**^	0.74^**^	0.48^*^	0.46^*^		0.58^**^	1	
PR_average 0.4–0.9_	−0.41^*^			0.71^**^	0.6^**^	0.63^**^	0.86^**^	0.81^**^	0.95^**^	0.92^**^	0.87^**^	0.65^**^	1
EC_a 0.0-0.1_	0.51^**^	0.35^*^			−0.37^*^				−0.56^**^	−0.56^*^	−0.55^*^		−0.44^**^
EC_a 0.1-0.2_	0.54^**^	0.51^**^	−0.35^*^	−0.41^**^	−0.53^**^			−0.53^**^	−0.64^**^	−0.67^**^	−0.67^**^	−0.43^**^	−0.46^**^
EC_a 0.2-0.3_	0.71^**^	0.72^**^			−0.42^**^			−0.63^**^	−0.63^**^		−0.68^**^	−0.39^*^	
EC_a 0.3-0.4_	0.75^**^	0.78^**^			−0.47^**^	−0.4^*^		−0.57^**^	−0.5^*^		−0.66^**^	−0.43^**^	
EC_a 0.4-0.5_	0.54^**^	0.52^**^		−0.46^**^	−0.64^**^	−0.49^**^	−0.55^**^					−0.51^**^	−0.5^**^
EC_a 0.5-0.6_	0.51^**^	0.57^**^		−0.46^*^	−0.57^**^	−0.49^**^	−0.64^**^	−0.6^**^			−0.52^*^	−0.5^**^	−0.54^**^
EC_a 0.6-0.7_		0.45^*^		−0.54^**^					−0.5^*^		−0.79^**^		−0.55^**^
EC_a 0.7-0.8_											−0.63^**^		
EC_a 0.8-0.9_													
EC_a average 0.0–0.4_	0.67^**^	0.61^**^		−0.38^*^	−0.53^**^			−0.58^**^	−0.69^**^	−0.68^**^	−0.72^**^	−0.43^**^	−0.44^**^
EC_a average 0.4–0.9_	0.57^**^	0.53^**^		−0.46^**^	−0.57^**^	−0.5^**^	−0.61^**^	−0.53^**^			−0.7^**^	−0.49^**^	−0.59^**^

	EC_veris 0.0-0.1_	EC_veris 0.1-0.2_	EC_veris 0.2-0.3_	EC_veris 0.3-0.4_	EC_veris 0.4-0.5_	EC_veris 0.5-0.6_	EC_veris 0.6-0.7_	EC_veris 0.7-0.8_	EC_veris 0.8-0.9_	EC_veris average 0.0–0.4_	EC_veris average 0.4–0.9_		

EC_a_-V													
EC_a_-H													
PR_0.0-0.1_													
PR_0.1-0.2_													
PR_0.2-0.3_													
PR_0.3-0.4_													
PR_0.4-0.5_													
PR_0.5-0.6_													
PR_0.6-0.7_													
PR_0.7-0.8_													
PR_0.8-0.9_													
PR_average 0.0–0.4_													
PR_average 0.4–0.9_													
EC_a 0.0-0.1_	1												
EC_a 0.1-0.2_	0.69^**^	1											
EC_a 0.2-0.3_	0.6^**^	0.71^**^	1										
EC_a 0.3-0.4_	0.53^**^	0.69^**^	0.95^**^	1									
EC_a 0.4-0.5_		0.62^**^	0.55^**^	0.7^**^	1								
EC_a 0.5-0.6_		0.53^**^	0.66^**^	0.77^**^	0.85^**^	1							
EC_a 0.6-0.7_		0.56^**^	0.53^**^	0.52^**^	0.59^**^	0.75^**^	1						
EC_a 0.7-0.8_		0.49^*^			0.55^*^	0.68^**^	0.93^**^	1					
EC_a 0.8-0.9_	0.62^*^	0.65^*^					0.71^**^	0.87^**^	1				
EC_a average 0.0–0.4_	0.83^**^	0.94^**^	0.86^**^	0.83^**^	0.61^**^	0.57^**^	0.56^**^		0.66^*^	1			
EC_a average 0.4–0.9_		0.53^**^	0.47^**^	0.61^**^	0.89^**^	0.96^**^	0.9^**^	0.9^**^	0.72^**^	0.55^**^	1		

**Table 3:** Fitted models and variogram parameters for all the variables measured by EM38 and VERIS P3000.

Variable	Geostatistical analysis	Model	*C*_0_	*C*_1_	*a* (m)
log⁡EC_a_-V	Universal kriging	Spherical	0.001	0.01	130.00
log⁡EC_a_-H	Universal kriging	Spherical	0.001	0.005	130.00
log⁡EC_a 0.0-0.1_	Universal kriging	Pure nugget effect			
log⁡EC_a 0.1-0.2_	Universal kriging	Pure nugget effect			
log⁡EC_a 0.2-0.3_	Universal kriging	Pure nugget effect			
log⁡EC_a 0.3-0.4_	Universal kriging	Pure nugget effect			
log⁡EC_a 0.4-0.5_	Universal kriging	Pure nugget effect			
log⁡EC_a 0.5-0.6_	Universal kriging	Pure nugget effect			
log⁡EC_a 0.6-0.7_	Universal kriging	Pure nugget effect			
log⁡EC_a 0.7-0.8_	Universal kriging	Pure nugget effect			
log⁡EC_a 0.8-0.9_	Universal kriging	Pure nugget effect			
log⁡EC_a average 0.0–0.4_	Universal kriging	Pure nugget effect			
log⁡EC_a average 0.4–0.9_	Universal kriging	Spherical	0.01	0.10	50.00
log⁡PR_0.0-0.1_	Universal kriging	Spherical	0.00	0.045	90.00
log⁡PR_0.1-0.2_	Universal kriging	Spherical	0.00	0.014	125.00
log⁡PR_0.2-0.3_	Universal kriging	Spherical	0.00	0.011	120.00
log⁡PR_0.3-0.4_	Universal kriging	Spherical	0.00	0.011	90.00
log⁡PR_0.4-0.5_	Universal kriging	Spherical	0.00	0.017	100.00
log⁡PR_0.5-0.6_	Universal kriging	Pure nugget effect			
log⁡PR_0.6-0.7_	Universal kriging	Pure nugget effect			
log⁡PR_0.7-0.8_	Universal kriging	Pure nugget effect			
log⁡PR_0.8-0.9_	Universal kriging	Pure nugget effect			
log⁡PR_average 0.0–0.4_	Universal kriging	Spherical	0.00	0.015	130.00
log⁡PR_average 0.4–0.9_	Universal kriging	Spherical	0.00	0.0095	70.00

*C*_0_: pure nugget effect; *C*_1_: structural variance; *a*: range.

**Table 4:** Fitted models and parameters of the cross-variograms between PR and EC_a_.

Variable	Geostatistical Analysis	Model	*C*_0_	*C*_1_	*a* (m)
log⁡PR_average 0.0–0.4_ × log⁡EC_a_-V	Universal cokriging	Spherical	0.00	0.0095	130.00
log⁡PR_average 0.0–0.4_ × log⁡EC_a_-H	Universal cokriging	Spherical	0.00	0.0065	130.00
log⁡PR_average 0.4–0.9_ × log⁡EC_a_-V	Universal cokriging	Spherical	0.00	0.0100	130.00
log⁡PR_average 0.4–0.9_ × log⁡EC_a_-H	Universal cokriging	Spherical	0.00	0.0075	130.00

*C*_0_: Pure nugget effect; *C*_1_: structural variance; *a*: range.

**Table 5:** Correlation coefficient (*r*) between measured and estimated data by kriging and cokriging for selected variables.

Universal Kriging		Universal Cokriging	
log⁡PR_average 0.0–0.4_	0.644	log⁡PR_average 0.0–0.4_ × log⁡EC_a_-V	0.676
		log⁡PR_average 0.0–0.4_ × log⁡EC_a_-H	0.696

log⁡PR_average 0.4–0.9_	0.339	log⁡PR_average 0.4–0.9_ × log⁡EC_a_-V	0.392
		log⁡PR_average 0.4–0.9_ × log⁡EC_a_-H	0.415

## Acronyms

EC_a_: Apparent soil electrical conductivity
EC_veris_: Soil electrical conductivity measured with penetrometer Veris P3000
PR: Soil resistance to penetration
EC_a_-H: Apparent soil electrical conductivity in horizontal dipole
EC_a_-V: Apparent soil electrical conductivity in vertical dipole
CV: Coefficient of variation.
